# Myopic Macular Retinoschisis in Teenagers: Clinical Characteristics and Spectral Domain Optical Coherence Tomography Findings

**DOI:** 10.1038/srep27952

**Published:** 2016-06-13

**Authors:** Chuan-bin Sun, Yong-sheng You, Zhe Liu, Lin-yan Zheng, Pei-qing Chen, Ke Yao, An-quan Xue

**Affiliations:** 1 Eye Center, Second Affiliated Hospital of Zhejiang University School of Medicine, Hangzhou 310009, China; 2 Eye Center, Zhejiang Provincial People’s Hospital, Hangzhou 310015, China; 3 Eye Hospital, Wenzhou Medical University, Wenzhou 325027, China

## Abstract

To investigate the morphological characteristics of myopic macular retinoschisis (MRS) in teenagers with high myopia, six male (9 eyes) and 3 female (4 eyes) teenagers with typical MRS identified from chart review were evaluated. All cases underwent complete ophthalmic examinations including best corrected visual acuity (BCVA), indirect ophthalmoscopy, colour fundus photography, B-type ultrasonography, axial length measurement, and spectral-domain optical coherence tomography (SD-OCT). The average age was 17.8 ± 1.5 years, average refractive error was −17.04 ± 3.04D, average BCVA was 0.43 ± 0.61, and average axial length was 30.42 ± 1.71 mm. Myopic macular degenerative changes (MDC) by colour fundus photographs revealed Ohno-Matsui Category 1 in 4 eyes, and Category 2 in 9 eyes. Posterior staphyloma was found in 9 eyes. SD-OCT showed outer MRS in all 13 eyes, internal limiting membrane detachment in 7 eyes, vascular microfolds in 2 eyes, and inner MRS in 1 eye. No premacular structures such as macular epiretinal membrane or partially detached posterior hyaloids were found. Our results showed that MRS rarely occurred in highly myopic teenagers, and was not accompanied by premacular structures, severe MDC, or even obvious posterior staphyloma. This finding indicates that posterior scleral expansion is probably the main cause of MRS.

Macular retinoschisis is one of the severe complications of high myopia which could lead to severe visual loss^1^,^2^,^3^,^4^,^5^. Although myopic macular retinoschisis (MRS) was first identified in 1999 by using optical coherence tomography (OCT), the initiation cause and pathogenesis of MRS remain unclear^1^,^2^,^3^,^4^,^5^. Most MRS was reported in older patients over 50, no report showed it occurred in teenager patients^1^,^2^,^3^,^4^,^5^,^6^,^7^,^8^.

Benhamou’s and our previous reports have revealed that MRS could occasionally occur in adult high myopes with mild myopic macular degenerative changes (MDC) without premacular structures such as macular epiretinal membrane or partially detached posterior hyaloids^2^,^9^. Therefore, we postulate that MRS might also be found in teenage patients who were usually lack of severe MDC or premacular structures. Our literature review based on Pubmed search with the keywords “macular retinoschisis, foveoschisis, myopic traction maculopathy, myopia” failed to identify any previous reports of MRS in teenagers.

OCT is an *in vivo*, non-invasive technology providing cross-sectional images of the retina that allows histopathology-like view of microstructures of each retinal layer. Compared with conventional time-domain OCT, spectral-domain OCT (SD-OCT) is much faster and more accurate in characterizing details of subtle macular changes. Therefore, it facilitates early detection of MRS^10^,^11^,^12^.

In this study, we retrospectively reviewed the medical records of highly myopic patients cared in our high myopia clinics, and identified MRS in 13 eyes of 9 teenagers based on its typical appearance in SD-OCT.

## Results

### Clinical characteristics of MRS in teenagers

Of the 2365 highly myopic patients retrospectively reviewed in this study, 228 cases (9.6%) were determined as MRS based on their typical appearance in SD-OCT, of whom 9 cases (0.38%) were teenagers. The demographic and clinical characteristics of 9 teenagers (13 eyes) identified in this study were shown in Table 1. There were 6 male (9 eyes) and 3 female (4 eyes) patients, the average age was 17.8 ± 1.5 years, range from 15 to 19 years. The average refractive error (spherical equivalent) was −17.04 ± 3.04 D, ranging from −12.13 D to −20.50 D. The average best-corrected visual acuity (BCVA) was 0.43 ± 0.61, ranging from 0.16 to 0.9. The average axial length was 30.42 ± 1.71 mm, ranging from 28.29 to 33.52 mm.

MDC evaluation by colour fundus photographs revealed Ohno-Matsui Category 0 in no eye, Category 1 in 4 eyes, Category 2 in 9 eyes, Category 3 and Category 4 in no eye. lacquer cracks were found in 2 eyes accompanied by Category 2 MDC. Typical posterior staphyloma by indirect ophthalmoscopy and B-type ultrasonography was found in 9 eyes but was not obvious in the other 4 eyes.

### SD-OCT findings of MRS in teenagers

**Figure 1:**
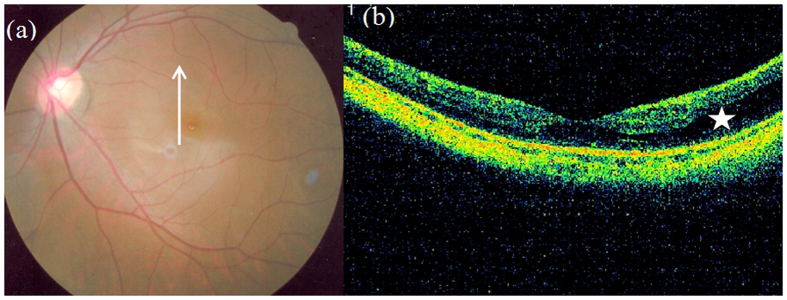
Outer macular retinoschisis (Fig.1(b), star) alone in an eye with a tessellated fundus but without posterior staphyloma (Case 6, left eye). The long arrow in Fig. 1(a) shows the direction of scanning, same in the following colour figures.

**Figure 2:**
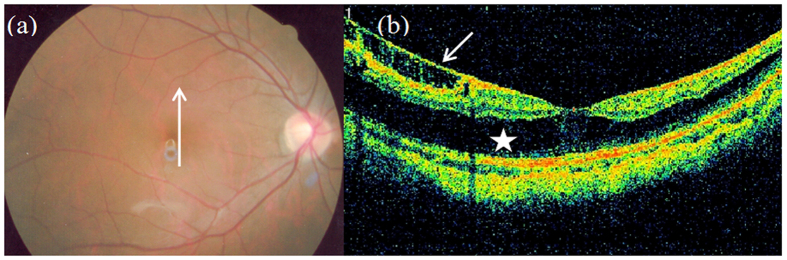
Outer macular retinoschisis (Fig.2(b), star) and internal limiting membrane detachment (Fig. 2(b), short arrow) in an eye with a tessellated fundus (Fig. 2(a)) but without posterior staphyloma (Case 6, right eye).

**Figure 3:**
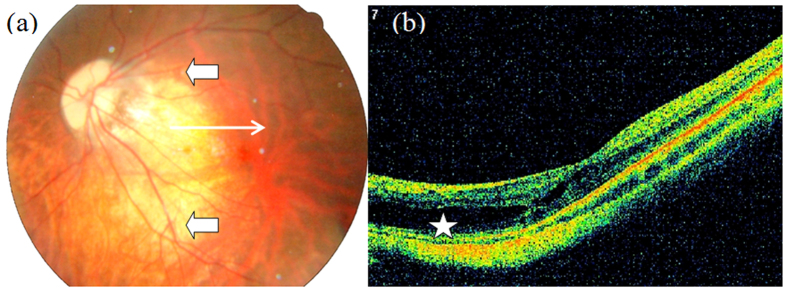
Outer macular retinoschisis (Fig.3(b), star) alone in an eye with diffuse chorioretinal atrophy and posterior staphyloma (Case 1). The arrow head in Fig. 3(a) shows the border of posterior staphyloma, same in the following colour figures.

**Figure 4:**
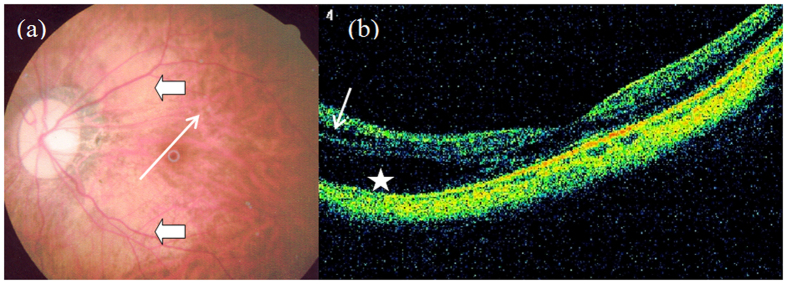
Outer (Fig.4(b), star) and inner (Fig. 4(b), short arrow) macular retinoschisis in an eye with diffuse chorioretinal atrophy and posterior staphyloma (Case 4, left eye).

**Figure 5:**
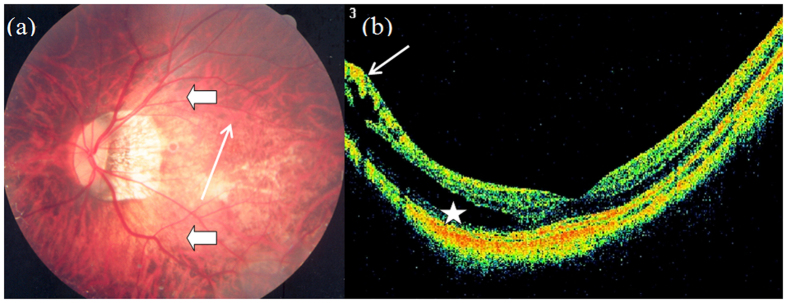
Outer macular retinoschisis (Fig.5(b), star) and vascular microfolds (Fig. 5(b), short arrow) combined with paravascular cyst in an eye with diffuse chorioretinal atrophy, lacquer cracks, and posterior staphyloma (Case 5, left eye).

**Figure 6:**
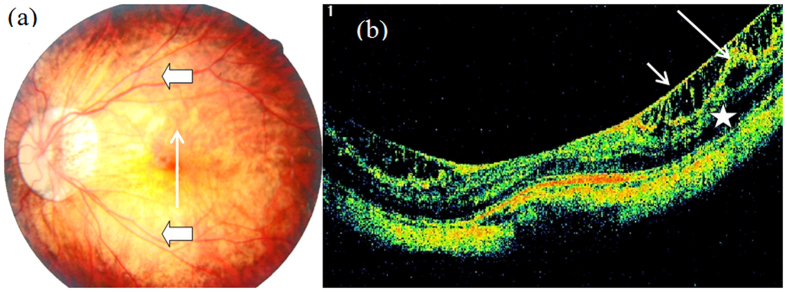
Outer macular retinoschisis (Fig.6(b), star) accompanied with internal limiting membrane detachment (Fig. 6(b), short arrow) and vascular microfolds (Fig. 6(b), long arrow) combined with paravascular cyst in an eye with diffuse chorioretinal atrophy, choroidal capillary atrophy and posterior staphyloma (Case 2, left eye).

Typical MRS was revealed by SD-OCT in 13 eyes of 9 highly myopic teenagers. Outer macular retinoschisis was found in all 13 eyes, internal limiting membrane (ILM) detachment was found in 7 eyes, vascular microfolds combined with paravascular cysts in 2 eyes, and inner macular retinoschisis in 1 eye, respectively (Figs 1, 2, 3, 4, 5, 6). Dome-shaped macula was found in 2 eyes. Neither premacular structures such as macular epiretinal membrane or partially detached posterior hyaloids, nor lamellar or full-thickness macular holes or macular detachment were found in our current cases. To evaluate the influence of posterior staphyloma on MRS formation in teenagers with high myopia, all cases were further divided into two groups according to whether or not obvious posterior staphyloma was found by indirect ophthalmoscopy and B-type ultrasonography (Table 2). There was a trend of better BCVA, shorter axial length, less severe spherical equivalent errors, and milder MDC in MRS eyes without posterior staphyloma compared to those with posterior staphyloma. The limited case number prevented meaningful statistical analyses.

## Discussion

MRS is not uncommon in highly myopic eyes^1^,^2^,^3^,^13^. However, MRS has not been reported in teenagers, and little is known about its clinical and morphological characteristics compared with MRS in older adult patients. Our finding that MRS can be detected in teenage high myopes is a novel finding. That MRS is not accompanied by premacular structures, severe MDC or obvious staphyloma has implications for the main cause and pathogenesis of MRS.

Our study revealed that MRS can occur, even though rarely, in teenagers with a percentage of 0.38% in our high myopia population, which was much lower than the percentage of MRS reported in older adult patients in literature (8–34%)^1^,^13^,^14^ and in general high myopia population in this study (9.6%). The two eye centers participating in this study are both the largest eye care facilities in their coverage areas, and are also the major referral centers for patients with eye diseases. Most patients with high myopia come to the eye centers for routine ophthalmic examination and refractive correction, and only a few high myopes present for uncorrectable visual loss or other ocular discomfort. Therefore, the percentage of MRS in teenagers reported in this study is likely representative of the true proportion of MRS in high myopia teenagers population.

MRS occurred most often in patients over 50 years in previous reports, and was usually accompanied with staphyloma, severe myopic macular atrophy and premacular structures such as macular epiretinal membrane or partially detached posterior hyaloids, which makes it difficult to identify what causes MRS^1^,^2^,^3^,^4^,^5^,^6^,^7^,^8^,^9^,^10^,^12^,^13^. A multivariate analysis showed that three factors were independently associated with foveoschisis in high myopia: axial length, macular chorioretinal atrophy, and vitreoretinal traction^14^. However, Benhamou’s and our previous reports have shown that there are adult MRS cases, although rare, without premacular structures or severe MDC. So we hypothesized that the main cause of MRS could be posterior scleral expansion. The coexistence of MRS and premacular structures, MDC, and even staphyloma might be parallel pathological changes of highly myopic eyes, since there is an increased incidence of staphyloma, MDC and premacular structures with age in highly myopic eyes. Posterior staphyloma and macular chorioretinal atrophy have already been reported to be secondary to posterior scleral expansion and the duration of high myopia^15^,^16^,^17^.

In this study, MRS was found to occur in highly myopic teenagers without premacular structures, severe MDC, or even obvious posterior staphyloma, which supports our hypothesis that posterior scleral expansion might be the initial pathological trigger of MRS in highly myopic eyes. Posterior scleral expansion can induce progressively outward expansion of choroid, retinal pigment epithelium, and neuroretina. However, the internal limiting membrane and retinal vessels can not be extended unlimitedly and, therefore, might induce an inward traction to the neuroretina soon after they have reached their expansion limits. Finally, the imbalance between the outward and inward traction results in the formation of macular retinoschisis^1^,^2^,^18^,^19^. The clinical finding that MRS most often occurs in the outer plexiform of the retina initially also supports our hypothesis.

A very interesting finding of our study is that 4 of 13 teenage eyes with MRS showed no obvious posterior staphyloma, which is different from previous reports of MRS in older adult patients. Posterior staphyloma is found in almost all patients over 50 years with MRS reported^1^,^2^,^3^,^4^,^5^,^6^,^7^,^8^,^9^,^12^,^13^. A possible explanation is that the posterior sclera of highly myopic teenagers preserves enough elasticity to counter a circumscribed protrution of posterior segment of an eyeball, which leads to a diffuse expansion of posterior sclera that is hardly found by either indirect ophthalmoscopy or even B-type ultrasonography. Our results showed that there was a trend of shorter axial length, better BCVA, less severe refractive error and milder MDC in MRS eyes without posterior staphyloma compared with those with posterior staphyloma in teenage high myopes. This is consistent with previous reports that posterior staphyloma usually occurs in highly myopic eyes with long axial length, severe refractive error and MDC. However, the sample size in this study was too small for meaningful statistical comparison between the two groups. Further study of larger volume is needed to evaluate above finding.

Another important finding of our study is that most MRS eyes in teenagers showed fairly good (≥0.3, 9/13 eyes) or good BCVA (≥0.8, 3/13 eyes), which was much better than the BCVA reported in MRS patients over 50 years^1^,^2^,^3^,^4^,^5^,^6^,^7^,^8^,^9^,^12^,^13^. Although it is natural that teenagers have a better BCVA than older adults, the different severity of MDC between teenagers and older adults with MRS may also contribute to their difference in BCVA. In contrast to severe MDC (Ohno-Matsui Categery 3 and Categery 4) frequently reported in patients over 50 years^1^,^2^,^3^,^4^,^5^,^6^,^7^,^8^,^9^,^12^,^13^, only mild (Ohno-Matsui Categery 1, 4/13 eyes) and moderate MDC (Ohno-Matsui Categery 2, 9/13 eyes) were found in teenage patients in our study. The milder MDC may contribute to the preserved oxygen and nutrient diffusion from choriocapillary layer to photoreceptors and inner neuroretina for their vitality and to maintain a fairly good visual acuity^20^.

In this study, most teenage MRS eyes (12/13) showed a fairly good to good BCVA and mild to moderate MDC but typical MRS appearance in OCT examination, which indicates that MRS can occur in highly myopic eyes without marked visual loss or severe MDC, and that OCT is a potent method for early detection of MRS in high myopes including teenagers. However, considering the very low percentage of MRS in highly myopic teenagers (no more than 0.38%), but high percentage (nearly 20%) of high myopia in Asian population, the routine use of OCT in teenager high myopes likely leads to an excessive increase in medical cost. We therefore recommend that OCT be performed for highly myopic teenagers with a decreased BCVA that can not be explained by refractive error or fundus examination.

In this study, we first report MRS occurring in teenage high myopes. Our study for the first time revealed that MRS could occur without premacular structures, severe MDC, or even obvious posterior staphyloma, which indicates that posterior scleral expansion is probably the main cause of MRS. This makes further progress for understanding the main cause and pathogenesis of MRS in high myopes.

## Methods

### Subjects

Subjects for this study were identified by a retrospective review of medical records in high myopia clinic of Eye Hospital, Wenzhou Medical University, and Eye Center, Second Affiliated Hospital of Zhejiang University School of Medicine, between October 2009 and December 2012. The medical data reviewed included colour fundus photography and OCT of 2365 highly myopic patients (age from 3 to 70 years). High myopia was defined as manifest refraction ≤−6 diopters and axial length ≥26 mm^21^,^22^. Based on the diagnostic criteria of macular retinoschisis in highly myopic eyes reported before^1^,^22^, MRS was determined in 228 cases according to its typical appearance in OCT examination, of whom 9 cases (13 eyes) were under 20 years of age.

All teenage patients identified in this study had complete ophthalmic examinations including spherical equivalent error, BCVA, slit lamp examination and indirect ophthalmoscopy, colour fundus photography, axial length measurement by IOL-Master, B-type ultrasonography, and SD-OCT. BCVA was measured in decimal but stated in logMAR format during statistical analysis. Posterior staphyloma was defined as a circumscribed or diffuse protrusion of the posterior sclera appearing in both indirect ophthalmoscopy and B-type ultrasonography. MDC were evaluated on basis of colour fundus photography in each participant and classified into five categories in accordance with myopic maculopathy classification and grading system reported by Ohno-Matsui *et al*. Category 0, normal-appearing fundus; Category 1: tessellated fundus; Category 2: diffuse chorioretinal atrophy; Category 3: patchy chorioretinal atrophy; Category 4: macular atrophy. In this system, three additional features, namely, lacquer cracks, myopic choroidal neovascularization, and Fuchs spot, were defined as “plus” lesions to supplement above categories. Posterior staphyloma was not recruited in this classification system^23^.

Institutional review board approvals were obtained from both Eye Hospital, Wenzhou Medical University, and Eye Center, Second Affiliated Hospital of Zhejiang University School of Medicine. The study was conducted according to the tenets of the Declaration of Helsinki. Informed consents were obtained from all participants.

### OCT Examination and Analysis

A SD-OCT machine RTVue-100 (Optovue, Fremont, CA) was used in this study. For each participant, RTVue-100 protocol MM6 was used to detect MRS. The MM6 protocol performed 12 radial line scans consisting of 1024 A-scans each (6 mm scan length) centered on the foveola with a total scan time of 0.27 seconds. The RTVue-100 examination followed the procedures reported in literature^10^,^11^,^12^. Only OCT images exactly centered on the foveola were chosen for analyses. MRS was defined as the splitting of intraretinal tissues including outer macular retinoschisis (outer plexiform layer splitting), inner macular retinoschisis (inner plexiform layer splitting), and internal limiting membrane detachment in macular area accompanied by either continuous or discontinuous bridging tissues in splitting cavities^1^,^2^,^3^,^4^,^5^,^6^,^7^,^8^,^9^. Patients with cystic cavities alone in the fovea in OCT images further underwent family survey to exclude X-linked hereditary juvenile retinoschisis^24^.

### Statistical Analysis

The average age, refractive error, BCVA and axial length were analyzed with SPSS 17.0.

## Additional Information

**How to cite this article**: Sun, C. B. *et al*. Myopic Macular Retinoschisis in Teenagers: Clinical Characteristics and Spectral Domain Optical Coherence Tomography Findings. *Sci. Rep.* **6**, 27952; 10.1038/srep27952 (2016).

## Figures and Tables

**Table 1:** Clinical and OCT characteristics of myopic macular retinoschisis in teenage high myopes.

No	Sex	Age	Eye	SER (D)	BCVA	AL (mm)	MDC	PS	Macular retinoschisis
1	M	19	L	−20.50	0.40	31.14	Category 2	+	ORS
2	F	19	R	−20.50	0.16	30.81	Category 2	+	ORS+ILMD
			L	−20.00	0.30	30.47	Category 2	+	ORS+ILMD+PVC
3	M	17	R	−18.00	0.3	30.04	Category 2	−	ORS+ILMD
4	M	16	R	−15.50	0.6	29.34	Category 2	+	ORS
			L	−16.63	0.4	30.57	Category 2	+	ORS+IRS
5	M	18	R	−20.26	0.4	34.1	Category 2+Lcs	+	ORS
			L	−19.38	0.5	33.52	Category2+Lcs		ORS+ILMD+PVC
6	M	18	R	−13.50	0.8	29.56	Category 1	−	ORSF+ILMD
			L	−12.50	0.9	29.2	Category 1	−	ORS
7	M	19	R	−15.63	0.3	29.33	Category 1	−	ORS+ILMD
8	F	19	R	−12.13	0.8	29.06	Category 2	+	ORS+ILMD
9	F	15	R	−17.00	0.4	28.29	Category 1	+	ORS

SER: spherical equivalent errors; BCVA: best corrected visual acuity (decimal format); AL: axial length; MDC: myopic macular degenerative changes; ORS: outer macular retinoschisis; IRS: inner macular retinoschisis; ILMD: internal limiting membrane detachment; Lcs: lacquer cracks; PS: posterior staphyloma; PVC: paravascular cyst.

^a^MDC classification: Category 0, normal-appearing fundus; Category 1: tessellated fundus; Category 2: diffuse chorioretinal atrophy; Category 3: patchy chorioretinal atrophy; Category 4: macular atrophy.

^b^Posterior staphyloma was defined as a circumscribed or diffuse protrusion of the posterior sclera appeared in both indirect ophthalmoscopy and B-type ultrasonography.

**Table 2:** Comparison of myopic macular retinoschisis with or without posterior staphyloma.

	Non-PS group	PS group
Eyes	4	9
Age (years)	18.0 ± 10.8	17.7 ± 1.6
Spherical equivalent errors (D)	−14.91 ± 2.44	−17.99 ±2.90
Best corrected visual acuity	0.50 ± 0.55	0.41 ± 0.63
Axial length (mm)	29.53 ± 0.37	30.81 ± 1.94
Myopic macular degenerative changes (MDC)		
Category 0	0	0
Category 1	3	1
Category 2	1	8
Category 3	0	0
Category 4	0	0

PS: posterior staphyloma.

^a^Posterior staphyloma was defined as a circumscribed or diffuse protrusion of the posterior sclera appeared in both indirect ophthalmoscopy and B-type ultrasonography.

^b^MDC classification: Category 0, normal-appearing fundus; Category 1: tessellated fundus; Category 2: diffuse chorioretinal atrophy; Category 3: patchy chorioretinal atrophy; Category 4: macular atrophy.
